# Isolation, Identification, and Antibiotic Susceptibility Testing of* Salmonella* from Slaughtered Bovines and Ovines in Addis Ababa Abattoir Enterprise, Ethiopia: A Cross-Sectional Study

**DOI:** 10.1155/2016/3714785

**Published:** 2016-08-29

**Authors:** Abe Kebede, Jelalu Kemal, Haile Alemayehu, Solomon Habte Mariam

**Affiliations:** ^1^Haramaya University College of Veterinary Medicine, P.O. Box 138, Dire Dawa, Ethiopia; ^2^Aklilu Lemma Institute of Pathobiology, P.O. Box 1176, Addis Ababa, Ethiopia

## Abstract

Salmonellae are ubiquitous, found in animals, humans, and the environment, a condition which facilitates transmission and cross contamination.* Salmonella enterica* serotypes exert huge health and economic impacts due to their virulence or carriage of antibiotic resistance traits. To address this significant issues with regard to public health, availability of adequate information on the prevalence and antibiotic resistance patterns of* Salmonella*, and establishment of adequate measures to control contamination and infection are needed. A cross-sectional study was conducted to assess the level of* Salmonella* infection in slaughtered bovines and ovines at Addis Ababa abattoir. Samples were collected randomly and processed for identification and antimicrobial susceptibility testing of* Salmonella* spp. From 280 animals examined, 13 (4.64%) (8 bovines and 5 ovines) were positive, with most samples (12/13, 92%) comprising* Salmonella* Dublin. Very high level of resistance to some antibiotics used in human medicine was detected. Most isolates were susceptible to gentamycin and amikacin. Nine (69%) of all isolates were resistant to multiple antibiotics. Serotyping revealed 12 of 13 isolates to be of the Dublin serotype with 9,12:g,p:- antigenic formula. This study emphasizes the importance of improving the evisceration practice during slaughtering and restricting the use of antibiotics in farm animals.

## 1. Introduction

Globally,* Salmonella* has been one of the most commonly reported causes of food-borne pathogens from distant and recent times [1–3]. According to a recent study [4] commissioned by the World Health Organization (WHO) on the global disease burden of food-borne diseases in humans, food-borne illnesses from diarrheal and invasive nontyphoidal* Salmonella enterica,* resulted in the largest disease burden, highlighting the significant public health importance of* Salmonella* infections and the urgency for control, particularly in low- and middle-income countries where most burden of diseases and occurrence of mortality cases are reported. In sub-Saharan Africa, nontyphoidal salmonellae are the most common causes of bacterial bloodstream infections in both adults and children presented with fever and are associated with case fatality rate of 20–25% [5]. Infections can occur most often via ingestion of contaminated meat, eggs, raw milk, fruits, and vegetables [6–8]. Contamination of these foods can occur during production, processing, distribution, and retail marketing [9]. Nontyphoidal salmonellae, including* S*. Dublin, are known to cause bacteremia and other infections in humans in sub-Saharan Africa [10–13].* S.* Dublin is primarily cattle-adapted, but it can also less frequently cause infections in other domestic animals, including ovines [14, 15]. Food-producing animals, including bovines and ovines, serve as reservoirs of nontyphoidal* Salmonella* serotypes that can be transmitted to humans [16, 17]. Wild animals can also serve as reservoirs of* Salmonella* increasing its transmittance to free-ranging food animals and then to humans through cross contamination [18, 19].

An increasing proportion of* Salmonella* isolates is resistant to commonly used antibiotics in both developing and developed countries [20, 21], and this increase is seen in both veterinary and public health sectors [22–24]. The increasing proportion of single and multiple antibiotic-resistant* Salmonella* strains isolated from human salmonellosis cases has been associated with the widespread use of antibiotics in food animals [25]. A recent review [26] indicated that, overall, several factors contribute to high antibiotic resistance in Ethiopia, including ease of access to and high frequency of antibiotic use, use of antibiotics at subtherapeutic levels, overprescription at health facilities, close contact between animals, high antibiotic use in animals in small production systems, and contamination during handling animal products; but another study indicated the exact extent of use of antimicrobials in food animals in Ethiopia is not clearly defined [25]. However, levels of antibiotics in beef have been found to be high in Ethiopia [27]. The antibiotics excessively used in Ethiopia and other African countries include tetracyclines, *β*-lactams, chloramphenicol, quinolones, nitrofurans, and macrolides. Tetracycline levels have been found to be especially high in meat and kidney samples from several abattoirs in Ethiopia, exceeding the WHO limits [27].

Contamination can occur at various levels. For example, fecal excretion of* Salmonella* can be a source of contamination both at farm and at abattoir levels. Contaminated hides and viscera can be sources of contamination at abattoirs. Abattoir workers can spread the contamination during evisceration and handling meat without proper hand disinfection. Some studies carried out on meat samples, minced meat, meat swab, and humans in Ethiopia showed that* Salmonella* is quite prevalent in various food animals (e.g., bovines, ovines, poultry, and pigs), animal products (e.g., beef, poultry, and milk), and human beings [28–32]. Animals and humans get* Salmonella* contamination in several ways. Animals get infected with* Salmonella* via the fecal-oral route through consumption of feeds, water, grass, and so forth, contaminated with feces from other infected animals, as well as through direct contact with infected animals.* Salmonella* can colonize animals at various sites, such as the intestines of food animals and the reproductive tract and egg of chicken, leading to contamination of various animal products. Humans become infected with* Salmonella* after consuming raw or improperly cooked animal products, such as contaminated meat, poultry, pork, and milk, as well as through direct contact with contaminated animals and household pets [16, 17, 33, 34].

In Ethiopia, as in other developing countries, it is difficult to evaluate the burden of food-borne diseases, because of the limited scope of studies and lack of coordinated epidemiological surveillance systems. In addition, underreporting of cases and the presence of other diseases considered to be of higher priority may have overshadowed the problem of food-borne diseases including salmonellosis. Therefore, the objectives of this study were to isolate and identify* Salmonella* in slaughtered bovines and ovines, estimate the prevalence, and investigate the susceptibility pattern of isolates to commonly used antibiotic agents using disk diffusion method.

## 2. Materials and Methods

### 2.1. Study Site Description and Duration

The study was conducted from December 2014 to April 2015 at Addis Ababa Abattoir Enterprise, Ethiopia. Addis Ababa is the capital city of Ethiopia. It lies in the central highlands at an altitude of 2324 m (7625 Ft) above sea level. The annual average maximum and minimum temperatures are 26°C and 11°C, respectively. The population size of the city is estimated at 3 million. The city's main supply of meat is provided by slaughter of bovines drawn from all corners of Ethiopia.

### 2.2. Bovine and Ovine Sampling

Healthy bovines and ovines were slaughtered at Addis Ababa abattoir enterprise after an average of 24–72 h upon arrival at the slaughterhouse. Feed and water were provided to the animals until 12 hrs before slaughter. Most of the bovines slaughtered at the abattoir are adult males of local zebu breed. Currently, on average, 1200 bovines and 1000 ovines are slaughtered every day. Different organs and carcasses are inspected after slaughter, mainly for presence of tuberculous lesions, whereas no tests are conducted to detect* Salmonella* and other pathogens.

### 2.3. Sample Size Determination

For isolation and identification of* Salmonella*, the sample size was calculated based on 8.5% [35] and 7.7% [36] expected prevalence in bovine and ovine samples, respectively, with 5% desired absolute precision and 95% confidence interval using the formula recommended by Thrusfield [37]:

(1) $$\begin{array}{c} n=\frac{Z^{2}\times p_{exp}\left(1-p_{exp}\right)}{d^{2}}, \end{array}$$

where *n* is required sample size, *Z* is 1.96, *p*_exp_ is expected prevalence, and *d* is desired absolute precision of 0.05.

Accordingly, the minimum sample size calculated was 120 and 109. To increase the precision of the estimate, the sample size was inflated and a total of 280 samples were considered. These samples included 140 from bovines (70 from carcass swab and 70 [35 each] from lung and liver) and 140 from ovines (70 from carcass swab and 70 [35 each] from lung and liver).

### 2.4. Study Design and Sampling Analysis

A cross-sectional study was conducted on randomly selected samples collected from Addis Ababa abattoir. Samples were collected using random sampling properly labelled by date of collection, sources, and sample type. The lung and liver samples were collected immediately after slaughter. Twenty-five grams of liver and lung samples was put in sterile plastic bags and kept in an ice box containing ice packs. The sampling areas were delineated by using sterile aluminum foil templates (10 × 10 cm). A sterile cotton tipped swab (2 × 3 cm) fitted with wooden shaft was first soaked in 10 mL of sterile buffered peptone water (BPW) (OXOID, England) and swabbed over the delineated area horizontally and then vertically several times on the abdomen (flank), thorax (lateral), crutch, and breast (lateral). Up on completion of the swabbing process, the swab was placed into the BPW within test tube and the upper tip of the shaft was broken and disposed of leaving the cotton swab [38]. The samples were kept in ice box containing ice packs and immediately transported to the Microbiology Laboratory at Aklilu Lemma Institute of Pathobiology. The samples were processed upon arrival.

### 2.5. Isolation and Identification

#### 2.5.1. Culture Methods, Plating, and Identification

The technique recommended by the International Organization for Standardization ISO 6579 [39] was employed in order to isolate and identify* Salmonella* organisms. Overnight frozen samples were allowed to thaw for 3 to 5 hrs at room temperature before analysis. The bacteriological media used for the study were prepared following the instructions of the manufacturer. Each 25 g of sample was put in a sterile Stomacher Bag and 225 mL of buffered peptone water (OXOID, England) was added (one to nine proportion) and homogenized using a laboratory blender (OXOID, England) for 2 minutes. The preenriched samples were incubated for 18 to 24 hrs at 37°C. Following this, 1 mL and 0.1 mL of the preenrichment broths were transferred aseptically into 10 mL of Muller Kauffmann Tetrathionate (Merck, Germany) and 10 mL of Rappaport-Vassiliadis (RV) broth (OXOID, England), mixed, and then were incubated for 18 to 24 hrs at 37°C and 42°C, respectively. Following incubation, a loop-full of each culture was streaked onto the surface of xylose lysine deoxycholate (XLD) (OXOID, England) and brilliant green agar (BGA) (OXOID, England) medium and incubated at 37°C for 24 to 48 hrs.

The XLD and BGA plates were examined for the presence of* Salmonella* colonies. If growth is slight or if typical colonies of* Salmonella* were not present, the plates were reincubated for a further 18 to 24 hrs and reexamined for the presence of typical* Salmonella* colonies. The formation of red colonies with black centers and of pink colonies with a red zone was inspected on XLD and BGA plates, respectively [40].

Identification of isolates was initiated by Gram staining. All Gram-negative isolates were further identified by motility test (by the hanging-drop method), biochemical tests, PCR amplification, and serotyping.

#### 2.5.2. Biochemical Tests

All suspected* Salmonella* colonies were picked from the agar plates and inoculated into the following biochemical test tubes for confirmation: triple sugar iron (TSI) test (presumptive* Salmonella* colonies produce black colonies or colonies with black centers and red medium on TSI agar) (OXOID, England), citrate test (presumptive* Salmonella* colonies produce blue colour for the citrate test), urease test (presumptive* Salmonella* colonies produce purple-red colour for the urease test), lysine decarboxylase (LDC) agar (OXOID, England) test (presumptive* Salmonella* colonies produce purple-coloured colonies on LDC agar), and indole test (presumptive* Salmonella* colonies produce violet-coloured colonies for the indole test). Plates were incubated for 24 or 48 hrs at 37°C [41]. Colonies were also tested for catalase production.

#### 2.5.3. Molecular Identification and Serotyping

A genus-specific polymerase chain reaction (PCR) amplification targeting histidine transport operon using pairs of primers 5′ACTGGCGTTATCCCTTTCTCTGGTG3′ and 5′ATGTTGTCCTGCCCCTGGTAAGAGA3′ was conducted to test the identity of the bacterial isolates using PCR conditions as described in Cohen et al. [42]. The bacterial isolates positive by the genus-specific PCR were serotyped by slide agglutination test targeting specific flagellar antigens.

### 2.6. Antibiotic Susceptibility Tests

The disc diffusion test was done for each isolate on Mueller-Hinton agar (OXOID, England). Approximately 20 mL of medium was poured into 90 mm diameter sterile Petri dishes to a depth of 4 mm and left at 37°C overnight to check for sterility. Five mL tryptic soya broth (OXOID, England) was inoculated with test isolates and incubated at 35°C for 4 hr. Culture of each isolate was compared with 0.5 McFarland turbidity standards (if necessary adjusted by adding sterile saline into tubes). Isolates were inoculated on Mueller-Hinton agar using swabs and inoculated plates were left at room temperature for 30 min to allow drying.* Salmonella* isolates were tested for susceptibility to the following 14 antibiotics (OXOID, England): amikacin (10 *μ*g), amoxicillin/clavulanic acid (30 *μ*g), ampicillin (10 *μ*g), ceftriaxone (30 *μ*g), chloramphenicol (30 *μ*g), ciprofloxacin (30 *μ*g), gentamycin (10 *μ*g), kanamycin (30 *μ*g), nalidixic acid (30 *μ*g), nitrofurantoin (30 *μ*g), streptomycin (10 *μ*g), trimethoprim/sulfamethoxazole, and tetracycline (30 *μ*g) using the disk diffusion method according to guidelines set by the Clinical Laboratory Standards Institute (CLSI) [43]. Antibiotic impregnated discs were dispensed on the surface of cultures of Muller-Hinton agar and incubated at 35°C for 20 hrs. The diameters of the zones of inhibition were recorded to the nearest mm and classified as resistant, intermediate, or susceptible according to established interpretive chart [43].

### 2.7. Quality Control

The following quality control strains were used:* Escherichia coli* ATCC 25922 for positive control and* Proteus mirabilis* ATCC 35659 for negative control in the LDC and indole tests;* Klebsiella pneumoniae* ATCC 700603 for positive control and* E. coli* ATCC 25922 and uninoculated for negative control in the citrate and urease tests; and* Salmonella typhimurium* ATCC 14028 were used.* K. pneumoniae* ATCC 700603 and* E*.* coli* were used as positive and negative controls, respectively, for the citrate tests. The sterility of prepared media was checked by incubating some randomly selected plates for 24 hrs at 37°C.* E. coli* ATCC 25922 and* K. pneumoniae* ATCC 700603 were used as positive and negative controls in the motility test.* E. coli* ATCC 25922 was used as a reference strain in the disk diffusion susceptibility tests.

### 2.8. Data Analysis

Data collected in the study and the results of laboratory investigations were entered into Microsoft Excel, edited, coded, and analyzed by statistical methods using statistical software program (SPSS version 15.0). Percentage to measure the prevalence of* Salmonella* was used and *P* value < 0.05 was considered significant and Chi-square test was used to compute the association between explanatory variables.

## 3. Results

### 3.1. Prevalence of* Salmonella* in Samples

Among a total of 280 samples examined for bacteriological status, 19 bovine and ovine samples were positive for* Salmonella* by biochemical testing (but the number of true positive samples was reduced to 13 by further tests as described in Section 3.2). The isolates from bovine and ovine samples were all Gram-negative rods and motile. The samples were positive for the citrate, LDC, and H_2_S production tests. The urease and indole tests were negative for these isolates. The isolates were positive for the catalase test.

### 3.2. Molecular and Serotype Identification of* Salmonella*

The PCR amplifications gave products of 496 bp for 13 isolates. This is the expected size for samples positive for* Salmonella* by the genus-specific PCR reaction applied here [42]. Serotyping also revealed the same 13 isolates to be* Salmonella* spp. Out of these positive samples, 8 (61.5%) were obtained from bovine samples and 5 (38.5%) were from ovine samples.* Salmonella* was isolated from 2 lung, 2 liver, and 4 carcass swab samples of bovines and from 1 lung, 3 liver, and 1 carcass swab samples of ovines (Table 1). Twelve of 13* Salmonella*-positive samples were found to be serotype Dublin and had the 9,12:g,p:- antigenic epitope for both bovine and ovine samples. The other* Salmonella* isolates of ovine origin had the I:Rough-O:g,p:- serotype (Table 2).

### 3.3. Antibiotic Susceptibility Tests

Single and multiple resistance to most of the antibiotics tested were observed. The highest level of resistance observed was streptomycin (100%) with all 13 isolates resistant to streptomycin. The next highest resistance was to amoxicillin, with 10/13 isolates (77%) being resistant. Gentamycin and amikacin were the most effective antibiotics, except that 1 (B.21) and 2 (B.S.23 and S.S.16) isolates were intermediate-resistant to the two antibiotics, respectively (Table 3). Ninety-two percent (12/13) of the isolates were found to be susceptible to gentamycin, while 85% (11/13) were susceptible to amikacin (Table 3 and see Supplementary Table S1 in Supplementary Material available online at http://dx.doi.org/10.1155/2016/3714785). The disk contents for all antibiotics we used were the same as those described under “Zone Diameter and Minimal Inhibitory Concentration (MIC) Interpretive Standards” in the CLSI document, except for amikacin and ciprofloxacin. For amikacin, we used 10 *μ*g disks and 11 of 13* S*. Dublin isolates were susceptible to the 10 *μ*g disk, while 2 were intermediate-resistant. This is in agreement with the indicated minimal inhibitory concentration (MIC), which is ≤16 *μ*g/mL. However, for ciprofloxacin, the disk content in the document is 5 *μ*g; we used 30 *μ*g disks, but only 3* S*. Dublin isolates were susceptible, while 10 were intermediate-resistant (Table 3). Indeed, the MIC of ciprofloxacin indicated in the document is ≤1 *μ*g/mL. Of all the isolates, 11 (85%) were considered to be multiple drug-resistant to two or more antibiotics (Table 3). No isolate was susceptible to all tested antibiotics.

## 4. Discussion

Studies on the prevalence and antibiotic susceptibility of* Salmonella* isolates from Addis Ababa abattoir are scarce. The presence of several* Salmonella* serotypes that can cause disease in both animals and humans, the high chance for zoonotic transmissions, and the fact that several antibiotic classes are used in both veterinary and human medicine dictate the need for continued surveillance of* Salmonella* in foods and the environment. This study found low-level contamination of sampled tissues and swabs with* S*. Dublin that, however, can be significant sources for further contamination during processing and handling.

Carcass contamination with* Salmonella* is of special public health significance for a country like Ethiopia, where consumption of raw and undercooked meat is common in most areas of Ethiopia. Contamination is likely to be further amplified as meat passes through various chains until it reaches the final consumers. A recent meta-analysis of* Salmonella* contamination in raw animal products in Ethiopia reported that *S*. Dublin was the most frequently isolated serotype in beef and revealed that the odds of contamination were >2-fold higher in retail markets than in abattoirs [44].

Although* S*. Dublin is known to be cattle-adapted, it can infect other domestic animals including ovines [14, 15, 45]. It primarily affects the mammary glands of cows and can be shed into milk subsequently causing infection in humans consuming unpasteurized dairy products [46–49]. Eguale et al. [50] reported the finding of* S*. Dublin in dairy cattle in Ethiopia. In Ethiopia, consumption of raw milk is very common largely because of the impracticability in most cases of boiling milk before consumption. Many people in Ethiopia also recognize the richness of liver in nutrients and consume it raw with hot pepper or spices. Thus, it is reasonable to assume that meat, milk, and liver can serve as vehicles for extensive transmission of* Salmonella* to humans.

*S*. Dublin can cause disease in animals and invasive disease and mortality in humans [51–53]. Nontyphoidal* Salmonella*, including* S*. Dublin, cause bacteremia, bloodstream, and other infections in humans in sub-Saharan Africa [10, 11]. Virulence plasmids are found in* S*. Dublin and other* Salmonella *serovars [54–56].* S*. Dublin causes enteritis by inducing infiltration of neutrophils into the intestinal epithelium, induction of inflammatory responses, and fluid secretion mediated by secretion systems that translocate secreted effector proteins into eukaryotic cells [52, 57].* S*. Dublin is considered highly pathogenic to humans, especially in immunocompromised individuals [12–14].

Resistance of bovine and ovine isolates of* Salmonella* to two or more major antibiotics was observed in 11 (84.6%) of isolates in this study. This resistance to antibiotics has significant importance because these antibiotics are also commonly used in human medicine in Ethiopia. For example, ciprofloxacin, nalidixic acid, and ceftriaxone are indicated against bacillary dysentery; chloramphenicol, trimethoprim/sulfamethoxazole, and ciprofloxacin are indicated against gastroenteritis; the latter two drugs are also used to treat cholera; amoxicillin, gentamycin, and ceftriaxone are among the agents used to treat pneumonia; trimethoprim/sulfamethoxazole and amoxicillin/clavulanic acid are used against sinusitis [58]. Moreover, prescriptions are usually made without prior isolation and drug susceptibility testing of infectious agents. Furthermore, the potential for horizontal transmission of resistance traits and other virulence factors or plasmids from* Salmonella* to other microbes, including within the human gut, may exist. Resistance traits in* Salmonella* can be genetically determined and may involve chromosomal mutations or may be plasmid-mediated and may be exchanged with other Enterobacteriaceae [59–62]. The magnitude of all these is not known in the prevailing conditions in Ethiopia since misdiagnosis and underdiagnosis can be common and prescriptions are virtually empirical. Large-scale intensive farming combined with use of antibiotics (which is empirical in most developing countries) in animals is expected to increase in the coming decades, thereby promoting on-farm selection of antibiotic-resistant strains and markedly increasing the human health risks associated with consumption of contaminated meat products [24, 25].

To our knowledge, this is the first study to have complete (100%) resistance to streptomycin in all studied isolates being reported among bovine or ovine isolates of* Salmonella* in Ethiopia. Thus, it is of significant concern, since our study involves all randomly selected samples. Resistance of* Salmonella* from food items, animals, and humans to streptomycin was reported by several studies in Ethiopia [35, 36, 50, 63–65], but the level of resistance to streptomycin ranged from 46 to 86%. This extremely high level of resistance of* Salmonella* and other pathogens (e.g., similar high level resistance to streptomycin has also been reported in Ethiopian tuberculosis patients, including in newly diagnosed patients ([66] and references therein)) to streptomycin in Ethiopia should be cause for high concern as it might also cause cross-resistance to other drugs with similar mechanism of action. Gentamycin was effective on almost all isolates in this study, similar to that of Garedew et al. [28] and Alemu and Zewde [67] who analyzed* Salmonella* from slaughtered bovines, raw meat, and swab samples from butcher shops' utensils and meat handlers.

This study has some limitations. Inclusion of possible* Salmonella* isolates from humans in the abattoir and the internal abattoir environment would have strengthened this study. The results of this study are indicative of* Salmonella* risk in meat from the abattoir, but more detailed studies should be conducted including possible routes of transmission of* Salmonella* as well as amplification of antibiotic resistance transfer.

## 5. Conclusions

The present study indicated detection of* Salmonella* from healthy slaughtered bovine and ovine samples at Addis Ababa abattoir enterprise with an overall prevalence of 4.64%. The results of the present study indicate poor evisceration process and hygienic practices of workers, which could result in the contamination of carcass and cross contamination from positive animals. This study also revealed high resistance of* Salmonella* to commonly used antibiotics. Contamination with* Salmonella* can be further amplified when one considers the possibilities for more contamination as meat passes through the many steps from slaughterhouse to the final consumer [29, 68]. Hygiene status must be enhanced to minimize cross contamination of* Salmonella* from utensils, cutting boards, and knifes as well as from abattoir workers who are involved in the slaughtering process. Even if* S*. Dublin were a bovine pathogen only, it would still be a highly significant problem to the economy and livelihood of people in general and farmers in particular. Proper decontamination and disinfection measures should be enhanced including at the entrance to slaughterhouses to reduce contamination. Further recommended measures to control contamination include carcass trimming to remove visible contamination, washes using ambient or hot water, organic acids, and other chemicals, as well as hide dehairing and frequent hand washes and disinfection. To minimize the risk of cross contamination and food-borne infections caused by* Salmonella* spp., control measures along the meat processing chain, namely, slaughterhouses, meat processing plants, distributors, and consumers, should be undertaken [69]. These measures should be introduced or practiced regularly at the Addis Ababa abattoir. Care should be taken in selecting antibiotics to treat* Salmonella* infection in animals and humans to avoid subtherapeutic levels of (appropriate) antibiotics and use of inappropriate antibiotics (e.g., antibiotic(s) to which an infectious agent may be resistant). Finally, larger studies are recommended to elucidate the magnitude of infection of animals with* Salmonella*, the extent of zoonotic transmissions, and the antibiotic resistance problem. A Pubmed search using combinations of key words such as “Addis Ababa”, “Abattoir”, “Salmonella”, “Bovine”, “Antibiotic Resistance” returned no published articles. We hope the results of this study could serve as basis for initiation of further and larger studies in Ethiopia that will shed light on the types, levels, frequency, and appropriateness of antibiotic use in farm animals as well as transmission of pathogens and antibiotic resistance from animals to humans, which will be important for the benefit of animal and human health. Such studies require implementation of carefully designed experiments to determine and trace associations between causes and effects and effects of subtherapeutic levels of antibiotics [70]. Thus, collaborative efforts with combined inputs of both expertise and resources would be needed.

## Supplementary Material

This Supplementary Table provides the susceptibility test results of the 13 *Salmonella* isolates to the tested antibiotics and gives the total numbers and percentages of isolates that are resistant or intermediate-resistant or susceptible to each tested antibiotic, and is a summary of the information presented in Table 3.

## Figures and Tables

**Table 1:** Prevalence of *Salmonella* based on species and sample type from Addis Ababa abattoir.

Sample	Bovine			Ovine			Total	Positive (%)	*χ* ^2^	*P* value
	Number examined	Number positive	Percent	Number examined	Number positive	Percent				
Carcass swab	70	4	5.7	70	1	1.4	140	5 (3.5)	1.37	0.50
Lung	35	2	5.7	35	1	2.8	70	3 (4.3)		
Liver	35	2	5.7	35	3	8.5	70	5 (7.1)		

Total	140	8	5.7	140	5	3.57	280	13 (4.64)		

**Table 2:** Serotype and antigen types of *Salmonella* isolates from bovine and ovine samples.

Species	Sample ID	Sample source	Serotype	Antigen
Bovine	B-17	Liver	Dublin	9,12:g,p:-
	B-21	Liver	Dublin	9,12:g,p:
	B-48	Lung	Dublin	9,12:g,p:
	B-61	Lung	Dublin	9,12:g,p:
	BS-1	Carcass swab	Dublin	9,12:g,p:
	BS-12	Carcass swab	Dublin	9,12:g,p:
	BS-13	Carcass swab	Dublin	9,12:g,p:
	BS-23	Carcass swab	Dublin	9,12:g,p:

Ovine	M-6	Liver	Dublin	9,12:g,p:
	M-26	Liver	Dublin	9,12:g,p:
	M-28	Liver	Dublin	9,12:g,p:
	M-72	Lung	I:ROUGH-O:g,p:-	-:g,p:-
	SS-16	Carcass swab	Dublin	9,12:g,p:

**Table 3:** Antibiotic disk diffusion susceptibility test results for bovine and ovine isolates of *Salmonella*.

Sample	Amik	Amox/clav	Amp	Ceftr	Chl	Cipro	Gent	Kan	Nal	Nitrof	Strep	Tri/sul	Tet
B.17	S	R	S	I	S	S	S	S	S	S	R	S	S
B.21	S	R	R	R	R	S	I	R	R	R	R	S	R
B.48	S	R	S	S	S	I	S	S	S	S	R	S	S
B.61	S	R	R	R	R	I	S	R	I	S	R	S	R
B.S.1	S	I	S	I	S	I	S	S	S	S	R	R	S
B.S.12	S	R	S	S	S	I	S	S	S	R	R	S	S
B.S.13	S	R	S	I	S	I	S	S	S	I	R	I	S
B.S.23	I	I	S	S	S	I	S	S	S	S	R	S	S
M.6	S	R	S	S	S	S	S	S	S	I	R	S	S
M.26	S	I	S	S	S	I	S	S	S	I	R	S	S
M.28	S	R	S	S	S	I	S	I	S	R	R	R	S
M.72	S	R	S	S	S	I	S	I	S	R	R	I	S
S.S.16	I	R	S	I	S	I	S	S	I	R	R	R	S

Amik: amikacin; Amox/clav: amoxicillin/clavulanic acid; Amp: ampicillin; Ceftr: ceftriaxone; Chl: chloramphenicol; Cipro: ciprofloxacin; Gent: gentamycin; Kan: kanamycin, Nal: nalidixic acid; Nitro: nitrofurantoin; Strep: streptomycin; Tri/sul: trimethoprim/sulphamethoxazole; Tet: tetracycline; S: susceptible; R: resistant; I: intermediate-resistant.
