# Supplementary material for: Isolation, Identification, and Antibiotic Susceptibility Testing of Salmonella from Slaughtered Bovines and Ovines in Addis Ababa Abattoir Enterprise, Ethiopia: A Cross-Sectional Study
Source: Int J Bacteriol. 2016 Aug 29;2016:3714785. doi: 10.1155/2016/3714785 (PMC5021890; doi:10.1155/2016/3714785)
Supplement: Supplementary file 1 — This Supplementary Table provides the susceptibility test results of the 13 Salmonella isolates to the tested antibiotics and gives the total numbers and percentages of isolates that are resistant or intermediate-resistant or susceptible to each tested antibiotic, and is a summary of the information presented in Table 3. [file 3714785.f1.docx]

Table S1. Summary of number of susceptible and resistant *Salmonella* isolates by tested antimicrobials (n=13).

| **Antimicrobial** | **Number of isolates** | | |
| --- | --- | --- | --- |
|  | **Resistant (%)** | **Intermediate (%)** | **Susceptible (%)** |
| Amikacin | - | 2(15) | 11 (85) |
| Amoxicillin/clavulanic acid | 10 (77) | 3(23) | - |
| Ampicillin | 2(15.4) | - | 11 (85) |
| Ceftriaxone | 2(15.4) | 4(31) | 7(54) |
| Chloramphenicol | 2(15.4) | - | 11(85) |
| Ciprofloxacin | - | 10(77) | 3(23) |
| Gentamycin | - | 1(7.7) | 12(92) |
| Kanamycin | 2(15.4) | 2(15.4) | 9(69) |
| Nalidixic acid | 1(7.7) | 2(15.4) | 10(77) |
| Nitrofurantoin | 5(38.4) | 3(23) | 5(38.4) |
| Streptomycin | 13(100) | - | - |
| Trimethoprim/Sulphamethoxazole | 3(23) | 2(15.4) | 8(61.5) |
| Tetracycline | 2(15.4) | - | 11(87) |
